# Comprehensive analysis of ferroptosis-related genes and prognosis of cutaneous melanoma

**DOI:** 10.1186/s12920-022-01194-z

**Published:** 2022-03-01

**Authors:** Changjiang Liu, Yuhang Liu, Yifeng Yu, Yong Zhao, Aixi Yu

**Affiliations:** Department of Orthopedics, Zhongnan Hospital of Wuhan University, Wuhan, 430071 Hubei People’s Republic of China

**Keywords:** Cutaneous melanoma, Ferroptosis, Overall survival, Biomarker, Immunity

## Abstract

**Background:**

Cutaneous Melanoma (CM) is a malignant disease with increasing incidence and high mortality. Ferroptosis is a new kind of cell death and related to tumor blood and lymphatic metastasis. This study aims at using bioinformatics technology to construct a prognostic signature and identify ferroptosis-related biomarkers to improve the prognosis and treatment of cutaneous melanoma.

**Methods:**

We used bioinformatics tools to analyze RNA sequencing expression data with clinical information from multiple databases, utilized varieties of statistical methods to construct a ferroptosis-related prognostic signature of cutaneous melanoma and screened out specific genes with independent prognostic ability.

**Results:**

We obtained 22 ferroptosis-related (*P* < 0.05) prognostic DEGs in the uniCox regression analysis, among which 10 high-expressed genes (ATG5, CHAC1, FANCD2, FBXL5, HMOX2, HSPB1, NQO1, PEBP1, PRNP, SLC3A2) were screened out by LASSO regression analysis to establish a predictive model. Meanwhile, the ferroptosis-related signature and the nomogram we drew performed an excellent performance based on Kaplan–Meier (K–M), Receiver operating characteristic (ROC) and calibration curves. Univariate and multivariable cox analyses displayed that our model was greater than other prognostic features. GO and KEGG analyses revealed that 10-biomarker signature was mainly related to epidermis differentiation and immunity. ssGSEA analysis indicated that the immune status between the two risk groups was highly different. Besides, we found that two genes (CP, ZEB1) had independent prognostic ability and can be applied for drug research. Both genes were highly related to immunity. GSEA illustrated that ZEB1 may be involved in cellular functions such as proliferation, apoptosis, and migration, while CP was closely connected to immune cell related functions.

**Conclusion:**

The present study suggested a 10-biomarker signature can be clinically used to predict the prognosis of cutaneous melanoma, which was better than conventional factors. CP and ZEB1 were independent prognostic genes and can be applied to guide treatment. In addition, ZEB1 mutation was highly related to overall survival in cutaneous melanoma, while CP may be associated with tumor progression. Our study comprehensively analyzed the relationship between iron metabolism, ferroptosis-related genes, and the prognosis of cutaneous melanoma, provided new insight for molecular mechanisms and treatment of ferroptosis and cutaneous melanoma.

**Supplementary Information:**

The online version contains supplementary material available at 10.1186/s12920-022-01194-z.

## Background

Cutaneous Melanoma (CM) is one of the most metastatic tumors caused by cells that produce pigments in the epidermis [1]. In 2020, Approximately 100,350 cases (5.6%) of newly diagnosed primary malignant tumors (excluding non-melanoma skin cancer) are skin melanoma and the incidence is still rising [2, 3]. Although melanoma only accounts for about 5% of skin cancers, it results in more than 75% of deaths [4]. Among the malignant tumors in the US in 2020, melanoma of the skin ranks 4th among men and 5th among women [2]. Current treatments for melanoma include surgery, targeted drugs and immunotherapy [5]. In addition, the five-year survival rate for melanoma with lymph node metastasis is 13–90%, and the prognosis is not greatly improved [6]. Therefore, there is an urgent need for a model to predict the prognosis of melanoma.

Ferroptosis was firstly proposed by Dixon in 2012 as a new type of programmed cell death [7]. Unlike traditional apoptosis, ferroptosis is iron-dependent and reactive oxygen-dependent. The main cytological change of ferroptosis is the decrease and disappearance of mitochondria instead of the characteristics, like cell swelling, in other cell deaths. The accumulation of intracellular iron makes cells sensitive to oxidative damage, and iron-dependent reactive oxygen species (ROS) will cause mitochondrial lipid peroxidation. Cells may die with the destruction of mitochondria [8–12]. Other research has shown that iron death plays an important role in liver cancer [13], lung cancer [14], colorectal cancer [15] and many other tumors by regulating a variety of tumor suppressor factors (including p53, BAP1) [16, 17]. It provides a good prospect for monitoring, diagnosing, predicting the prognosis of the disease. Meanwhile, a recent study showed that ferroptosis can inhibit blood and lymphatic metastasis of the tumor [18]. Therefore, analyzing the correlation between iron death-related genes and the prognosis of tumor patients is reasonable and important.

At present, there are studies exploring prognostic gene models related to ferroptosis in tumors. Liu et al. demonstrated that a ferroptosis-related prognostic model can be used to predict the survival rate of patients with glioma [19]. Liang et al. [20] constructed a prognostic genetic model of hepatocellular carcinoma related to ferroptosis. Luo et al. found that iron death-related genes can be used to predict the prognosis of uveal melanoma [21]. In order to explore the relationship between ferroptosis and melanoma patients survival status and increase the choices in the diagnosis and treatment of melanoma and the prognostic judgment, we used data from databases including TCGA, GTEx, GEO and previous studies to analyze iron death-related genes in melanoma and construct a ferroptosis-related gene model that can be employed for prognosis prediction. Ferroptosis-related genes in prognostic model will promote new ideas for the exploration of tumor pathogenesis and further treatment.

## Methods

### Data collection

In the current study, a total of 1284 RNA sequencing (RNA-seq) data and clinical materials about cutaneous melanoma (CM) were included in the training group. Among them, 472 samples were downloaded from the TCGA database,^1^ including 1 normal and 471 tumor samples. To increase the normal sample size, we collected the expression profiles of 812 normal skin samples in the GTEx data downloaded through UCSC Xena^2^ [20, 22]. Subsequently, an expression matrix file (GSE65904) from the GEO Database^3^ was used as external verification. All data are publicly available. Therefore, this study was exempt from the permission of the local ethics committee.

### Acquisition of genes related to ferroptosis and iron metabolism

Genes related to ferroptosis were obtained in the iron death pathway (map04216) in the KEGG Database^4^ [23]. Genes related to iron metabolism and cellular iron ion homeostasis were derived from the iron uptake and transport pathways (R-HSA-917937) in the Reactome Pathway Database^5^ and the AmiGo2 Database,^6^ respectively [24]. We eliminated the duplicate ones after adding ferroptosis genes reported recently [20] and thus a total of 173 iron death-related genes were used in this study. The whole genes list was displayed in Additional file 2: Table S1.

### Data processing in training and validation cohorts

In the TCGA and GTEx cohorts, genes expression levels were reannotated by the "rtracklayer" R package and batch effects were eliminated by "sav" R package. We applied "affy" R package to use the Robust Multi-Array Average (RMA) method to normalize data. DEGs were distinguished by using the "limma" R software package with a false discovery rate (FDR) < 0.01. Then, univariate Cox regression analysis of overall survival (OS) was conducted to identify iron death-related genes with prognostic value. We took the intersection of DEGs and the ferroptosis-related prognostic genes. Heatmap and box-line map were drawn by the "pheatmap" and "ggpubr" R software packages, which could visually display the expression of genes in two groups. Prognostic-related DEGs interaction network analysis was generated in the STRING Database^7^and GeneMANIA databases^8^ [25]. Then we performed enrichment analysis in the Metascape database.^9^ To make these genes have the same prognostic prediction orientation, we divided them into two groups: high-and low-expressed groups in tumors. We used the "glmnet" R software package to carry out LASSO-penalized Cox regression analysis on high-expressed genes to avoid over-fitting of the model [20, 26]. The regressed genes were used to construct a risk scoring model. The risk score of each melanoma patient was calculated through a scoring formula (Risk score = $\sum_{i}^{n}{\mathrm{Exp }}_{i}* {\beta }_{i}$; n, ${\mathrm{Exp }}_{i}$, and ${\beta }_{i}$ represent the number of genes, gene expression level, and regression coefficient value, respectively). melanoma patients were parted into high- and low-risk groups according to the median risk score. Meanwhile, we divided the patients into low-stage group (Stage I/II) and high-stage group (Stage III/IV) according to AJCC. Next, we used the "survminer" and "survivalROC" R software package to draw Kaplan–Meier (KM) survival curves for the two groups. Receiver operating characteristic curves (ROC) were applied to predict the sensitivity and specificity of the prognostic model. In addition, the "stats" R package was used for principal component analysis (PCA) to explore the distribution of different groups based on the survival status. Then, we used GEO data to test and verify these results. Univariate Cox analysis and multivariate Cox analyses were used to judge whether the risk scoring model and other parameters can be regarded as independent prognostic factors. To increase the predictability of the risk score model, we integrated other features of melanoma patients and a nomogram was drawn by the "rms" R package to predict OS in melanoma patients. Calibration curves at 3-, 5- and 10-year were drawn to evaluate the difference between the observed results and the nomogram-predicted results.

### Functional enrichment analysis

All DEGs between high-risk and low-risk groups were identified by using the "limma" R software package (|log2FC|≥ 1, FDR < 0.05). Next, we used "Cluster Profiler" R software package to carry out Kyoto Encyclopedia of Genes and Genomes (KEGG) and Gene Ontology (GO) analyses on DEGs (*P* < 0.05) [27, 28]. The single gene enrichment analysis (ssGSEA) was conducted by "gsva" R software package to calculate the activity of 13 immune-related pathways and the infiltration score of 16 immune cells (Additional file 3: Table S2).

### Independent prognostic analysis of ferroptosis-related genes

We performed univariate Cox analysis and multivariate Cox analysis to clarify the independent prognostic ability of the genes in the high- and low- expression group, respectively. Then we screened out the genes that had independent prognostic ability in both univariate and multivariate Cox analyses. These genes will be used in the next part.

### GEPIA and PrognoScan

Based on the independent prognostic genes, we compared the mRNA expression of these genes in 461melanoma tissues and 558 adjacent tissues (log2FC |≥ 1, *P* < 0.05) and drew the survival curves of genes with overall survival time and disease-free survival (DFS) time in Gene Expression Profiling Interactive Analysis (GEPIA) databases,^10^ which is an online tool. Then we again verified them in the PrognoScan database,^11^ which is a tool for assessing the biological relationship between gene expression and prognosis by employing the minimum P-value approach for grouping patients for survival analysis [29].

### cBioPortal

cBioPortal database^12^ is a resource-rich website, providing visualization tools for research and analysis of cancer genetic data [30]. In order to understand the relationship between the genomics alternations of these genes and melanoma, we analyzed the alternation manifestation and relationship between gene mutation and survival time in cBioPortal database and drew K–M curves of genes alternation groups and non-mutated groups.

### TISIDB and TIMER

TISIDB^13^ is an online database containing a large amount of tumor immunity related data, which can be utilized to analyze the relationship between genes and immune, clinical information and drugs [31]. We used this database to gain a comprehensive understanding of a gene and its association with survival in various tumors, including cutaneous melanoma. And we further explored the correlation between these genes and the infiltration of 6 major immune cells (B cells, CD4+ T cells, CD8+ T cells, macrophages, dendritic cells and neutrophils) in the TIMER^14^ database.

### GSEA

Based on genomics alternations of these genes, we identified that the signaling pathways are differentially activated in these genes. We divided melanoma patients into high-risk and low-risk groups according to single gene expression. Then we performed Gene set enrichment analysis (GSEA) on each gene. Gene set permutations were performed 1000 times for each analysis. *P* value < 0.05 and *q* value < 0.25 were considered as significant.

### HPA

The HPA database^15^(Human Protein Atlas) is a website about proteomics, transcriptomics and systems biology data, which can map tissues, cells, organs, etc.

### Statistical analysis

All data were analyzed with R 3.6.3 software. Student's t-test was applied to identify the DEGs between non-tumor tissues and tumor tissues. The chi-square test was applied for the comparison of proportional differences. The adjustment of the P value was abided by Benjamini & Hochberg (BH) method. Kaplan–Meier analysis and log-rank test were carried out to compare OS between two groups. In addition, univariate and multivariate analyses were used to identify independent prognostic factors of OS. If not specified above, a *P* value less than 0.05 was considered statistically significant.

## Results

There were 471 melanoma patients and 813 normal samples finally enrolled from the TCGA-SKCM cohort and GTEx cohort.

### Identification of prognostic ferroptosis-related DEGs

**Fig. 1:**
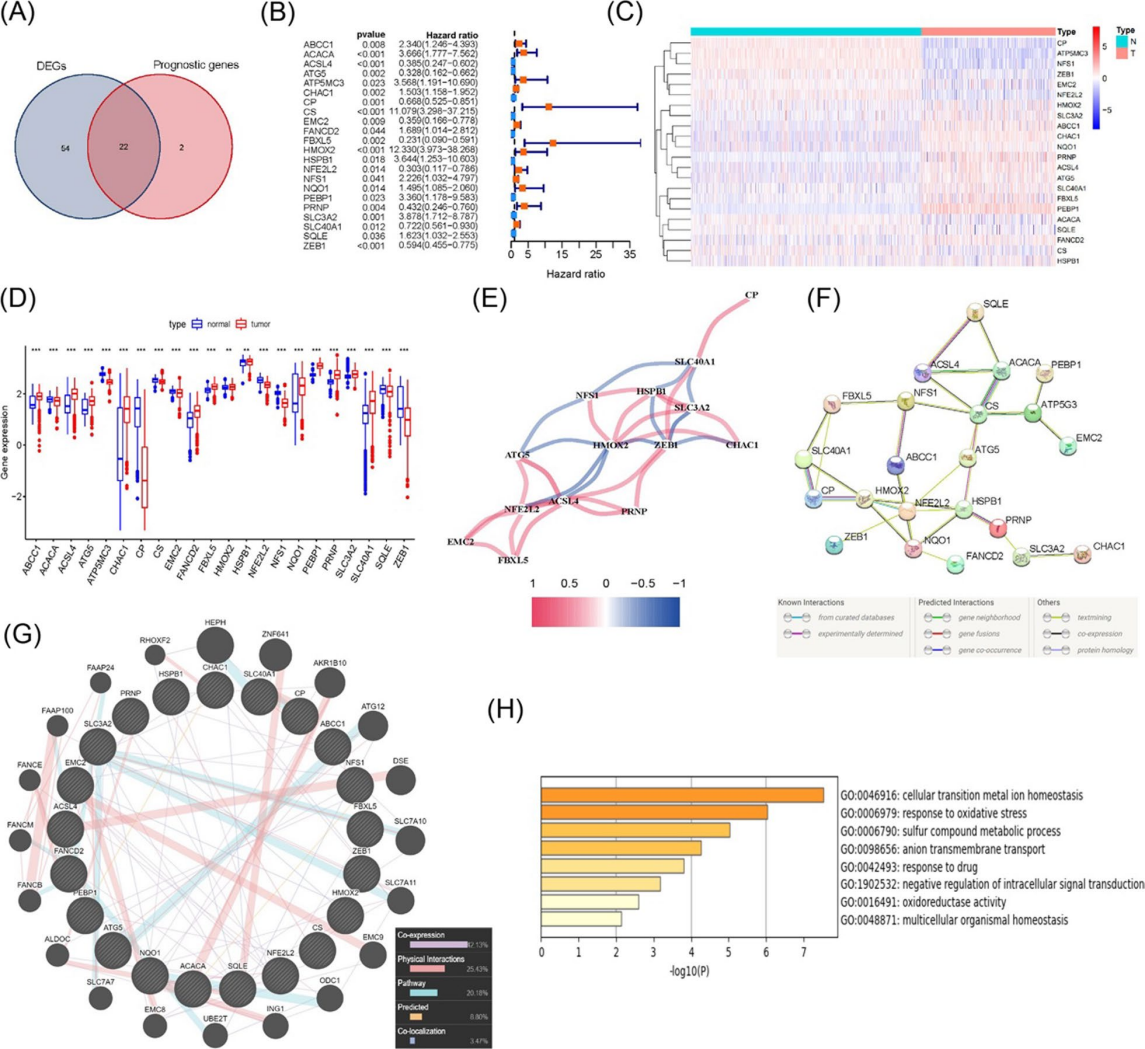
Identification of prognostic ferroptosis-related DEGs in the GTEx and TCGA cohort. **A** Venn-diagram to obtain survival-related genes that were differentially expressed in tumor and adjacent non-tumor tissues. **B** Forest-plots displaying the 22 genes of the uniCox regression analysis in relationship with OS and gene expression. **C** Heatmap showing the expression of 22 overlapping genes in a single tissue. **D** Boxplot showing the difference in expression of intersection genes in tumor and normal tissues. **E** Interactive network displaying the relevance of 22 genes in TCGA cohort. **F**, **G** Correlation heatmap in STRING database and GeneMANIA database, respectively. **H** GO enrichment analysis of 22 genes in Metascape database

A total of 76 iron death-related genes (76/173, 43.9%) were differentially expressed in non-tumor tissues and tumor tissues. 76 iron-death genes were considered DEGs and 24 prognostic-related genes were related to OS in the uniCox regression analysis (Additional file 4: Table S3 and Additional file 5: Table S4). After calculating the intersection, 22 prognostic ferroptosis-related genes were included in the study (Fig. 1A, B). Heatmap and boxplot showed that there were 13 highly expressed genes in tumor samples (Fig. 1C, D). The related link of 22 genes in our cohort was shown in Fig. 1E. HMOX2, ZEB1, HSPB1, SLC40A1, NFE2L2 and ACSL4 seemed to be the hub genes, where HMOX2 and ZEB1 were negatively related to many genes. The correlation analysis network diagram in the STRING database illustrated that CS, CP, NFE2L2, HMOX2, and HSPB1 were the key genes (Fig. 1F), which was similar with the results in our cohort. The correlation analysis network diagram in GeneMANIA database showed that there was a high co-expression rate (42.13%) and physical interaction (25.43%) among these genes (Fig. 1G). In the Metascape database, GO enrichment analysis results indicated that these genes were mainly related to the cellular transition metal ions homeostasis and the response to oxidative stress (Fig. 1H). We also found that some genes can be divided into two groups, high expression genes (ABCC1, ACSL4, ATG5, CHAC1, FANCD2, FBXL5, HMOX2, HSPB1, NQO1, PEBP1, PRNP, SLC3A2, SLC40A1, SQLE) and low expression genes(ACACA, ATP5MC3, CP, CS, EMC2, NFE2L2, NFS1, SQLE, ZEB1). When these low-expressed genes were used to calculate risk score with high-expressed genes, the weighted total score may be affected [20]. Consequently, we chose the high-expressed genes and performed LASSO regression analysis. When λ value was optimal, 10 genes (ATG5, CHAC1, FANCD2, FBXL5, HMOX2, HSPB1, NQO1, PEBP1, PRNP, SLC3A2) were identified to be utilized in the prognostic model.

### Prognostic value of the 10 ferroptosis-related genes signature in TCGA cohort

**Fig. 2:**
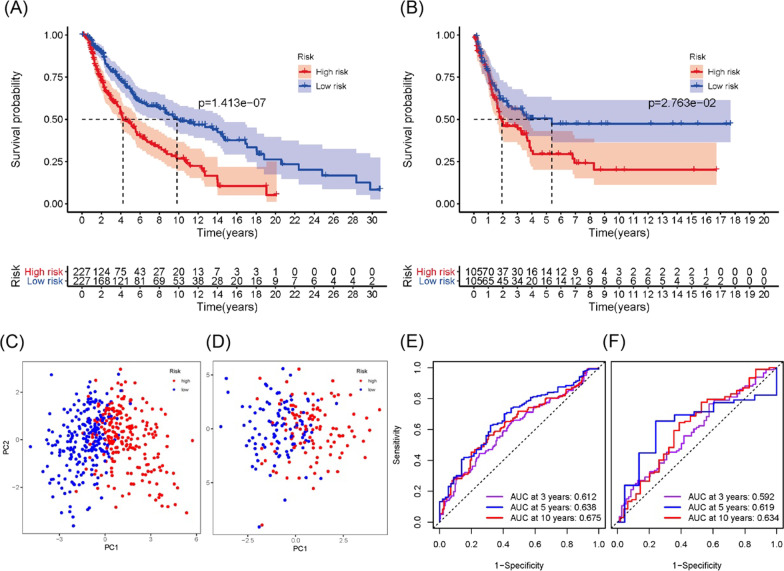
Prognostic analysis of a 10-biomarker signature in TCGA cohort and GEO matrix. **A**, **B** Kaplan–Meier curves for two different-risk melanoma patient groups in TCGA cohort and GEO matrix, respectively. **C**, **D** PCA plots of the melanoma patients in TCGA cohort and GEO matrix, respectively. **E**, **F** the ROC curves of 3, 5, and 10 years of survival rates using 10 genes prognostic model in TCGA cohort and GEO matrix, respectively

According to the scoring formula above, we calculated the risk value of each melanoma patient and divided them into high-risk group (n = 227) and low-risk group (n = 227) on the basis of the median cut-off value. The Kaplan–Meier curve illustrated that the overall survival time of the two groups had a significant difference (Fig. 2A, *P* < 0.001). PCA analysis could clearly reveal that patients were scattered in two different directions (Fig. 2C). The time-dependent ROC curve can evaluate the predictive ability of the prognostic model for survival. It demonstrated the value of the area under curve (AUC) at 3-, 5- and 10-year OS in this prognostic model were 0.612, 0.638, 0.675, respectively (Fig. 2E). Meanwhile, in order to access the value of our signature in predicting the prognosis of melanoma in different stages, we divided the TCGA data into two groups (185 for stage I/II and 154 for stage III/IV) to further validation. The K-M curve showed that patients with high risk scores had worse prognosis (Additional file 1: Fig. S1A and Fig. S1C). The AUC values of 3, 5, and 10 years in the ROC curves were 0.608, 0.594, 0.668 in the stage I/II and 0.629, 0.654, 0.793 in the stage III/IV, respectively (Additional file 1: Fig. S1B and Fig. S1D). PCA analysis outcomes were in according with previous study (Additional file 1: Fig. S1E and Fig. S1F). We performed the same analysis in primary tumors and metastatic melanoma. The results showed that in metastatic melanoma, high-risk patients had a worse prognosis (*P* < 0.001), which was not statistically significant in primary tumors (Additional file 1: Fig. S2A and S2C). The ROC curves indicated that in metastatic melanoma, our AUC values were in the range of 60–68% (Additional file 1: Fig. S2B), whereas in primary tumors there was no statistical analysis. PCA analysis illustrated that high-risk and low-risk patients were in two directions (Additional file 1: Fig. S2E and Fig. S2F).

### Evaluation of the 10 ferroptosis-related genes model in the GEO

To confirm the reliability of 10 ferroptosis-related genes model, we applied the same formula to calculate the risk score of each patient in the GEO database, and divided the patients into a high-risk group and a low-risk group by risk value. Meanwhile, the K-M curve also showed that the patients at high-risk had a longer survival period than the ones at low-risk (Fig. 2B). Similarly, PCA analysis displayed that melanoma patients in the two groups were scattered in distinct directions (Fig. 2D). In addition, the AUC values of the time-dependent ROC curves were 0.592, 0.619, 0.634 in the prognosis model of these 10 biomarkers (Fig. 2F).

### Independent prognostic value of the 10-gene signature

**Fig. 3:**
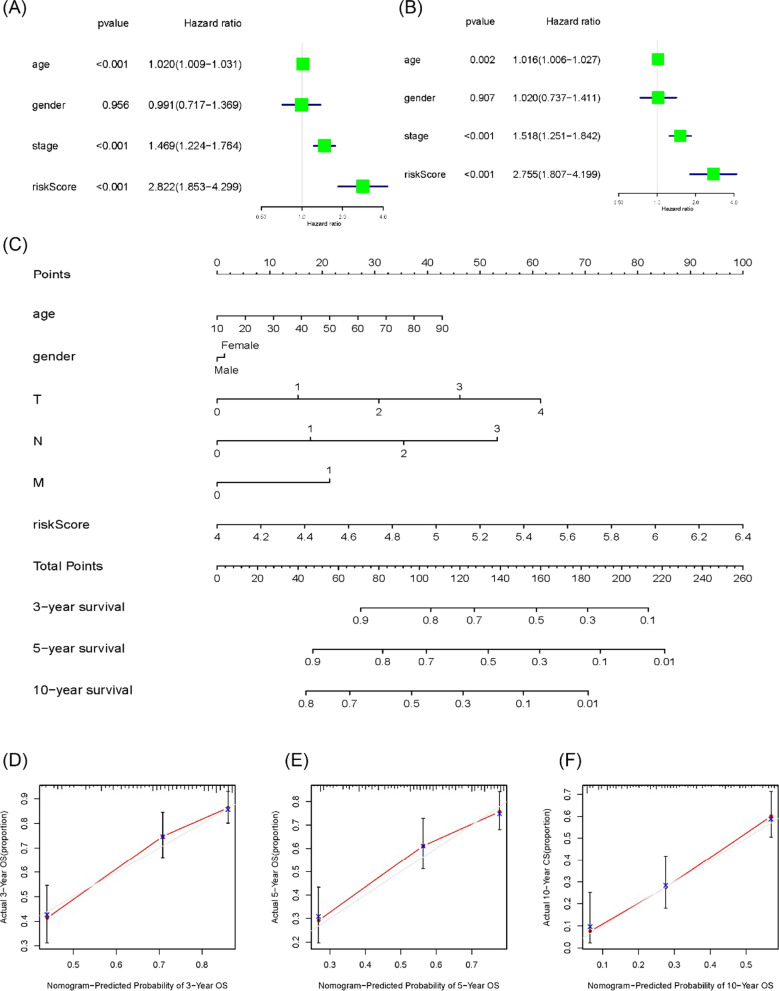
Independent prognostic analysis of 10-gene risk scores and a nomogram. **A** The univariate Cox regression analysis of risk scores with other parameters. **B** Multivariate Cox regression analysis. **C** Nomogram used to predict 3, 5, and 10-year survival rates of melanoma patients. **D**–**F** Calibration curves of the nomogram at 3, 5, and 10 years

Univariate and multivariate Cox regression were conducted to analyze TCGA cohort data to evaluate whether clinical parameters and risk scores were independent prognostic factors for OS. It demonstrated that the risk score was highly related to OS in both univariate and multivariate Cox analyses and can be applied as an independent prognostic indicator (Univariate: HR = 2.822, 95% CI = 1.853–4.299, *P* < 0.001; Multivariate: HR = 2.755, 95% CI = 1.807–4.199, *P* < 0.001; Fig. 3A, B). The ROC curves illustrated that our signature for prognostic prediction was better than other covariates with AUC value of 0.685 (Additional file 1: Fig. S3). Moreover, we drew a nomogram that could be utilized to predict the survival rate at 3-, 5-, and 10-year. The strategy to predict a patient’s prognostic status was to calculate the corresponding score and total score which corresponded to the different prognostic survival rates based on the patient’s clinical information and risk score (Fig. 3C). The calibration curves of 3-, 5-, 10-year proved that the nomogram-predicted results were consistent with the patient's actual survival data, demonstrating a valid and valuable prognostic model (Fig. 3D–F).

### Functional enrichment analyses

**Fig. 4:**
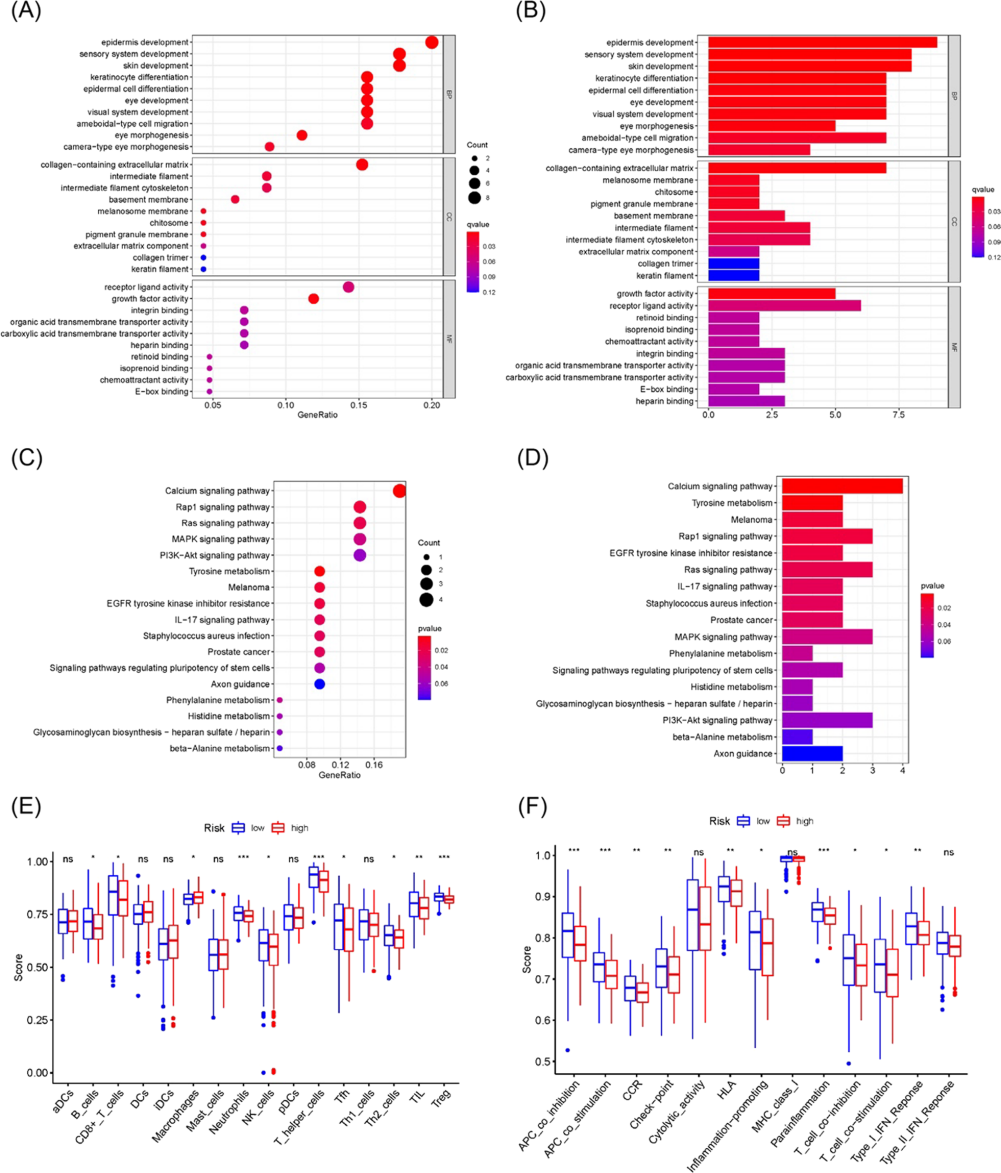
The enrichment analyses of GO, KEGG and ssGSEA in TCGA cohort. **A**, **B** GO enrichment analysis of DEGs in high- and low-risk group. **C**, **D** Results of KEGG enrichment analysis. **E**, **F** Results of ssGSEA enrichment analysis in the infiltration score of 16 immune cells and the activity of 13 immune-related pathways

To figure out the biological pathways and functions concerning genes in the prognostic model, we identified 52 DEGs in two groups, and performed GO enrichment and KEGG pathway analyses (Additional file 6: Table S5). It elucidated that DEGs were largely enriched in epidermis development, collagen-containing extracellular matrix, and growth factor activity (Fig. 4A, B). The complete GO enrichment results were presented in Additional file 7: Table S6. KEGG functional enrichment analysis displayed Calcium signaling pathway, Rap1 signaling pathway, Ras signaling pathway, MAPK signaling pathway, PI3K − Akt signaling pathway (Fig. 4C, D). The complete KEGG enrichment results were presented in Additional file 8: Table S7. We then conducted ssGSEA in different immune cell subgroups and related functions to further explore the interrelation between genes and immune status in the risk model. In the two groups, Neutrophils and Treg cells were significantly different (*P* < 0.001), and most immune-related functions were lower enriched in melanoma patients at high risk. These indicated that the inflammatory infiltration in tumor sites was negatively correlated with the melanoma patient’s prognosis (Fig. 4E, F).

### Independent prognostic analysis of all ferroptosis-related genes

**Fig. 5:**
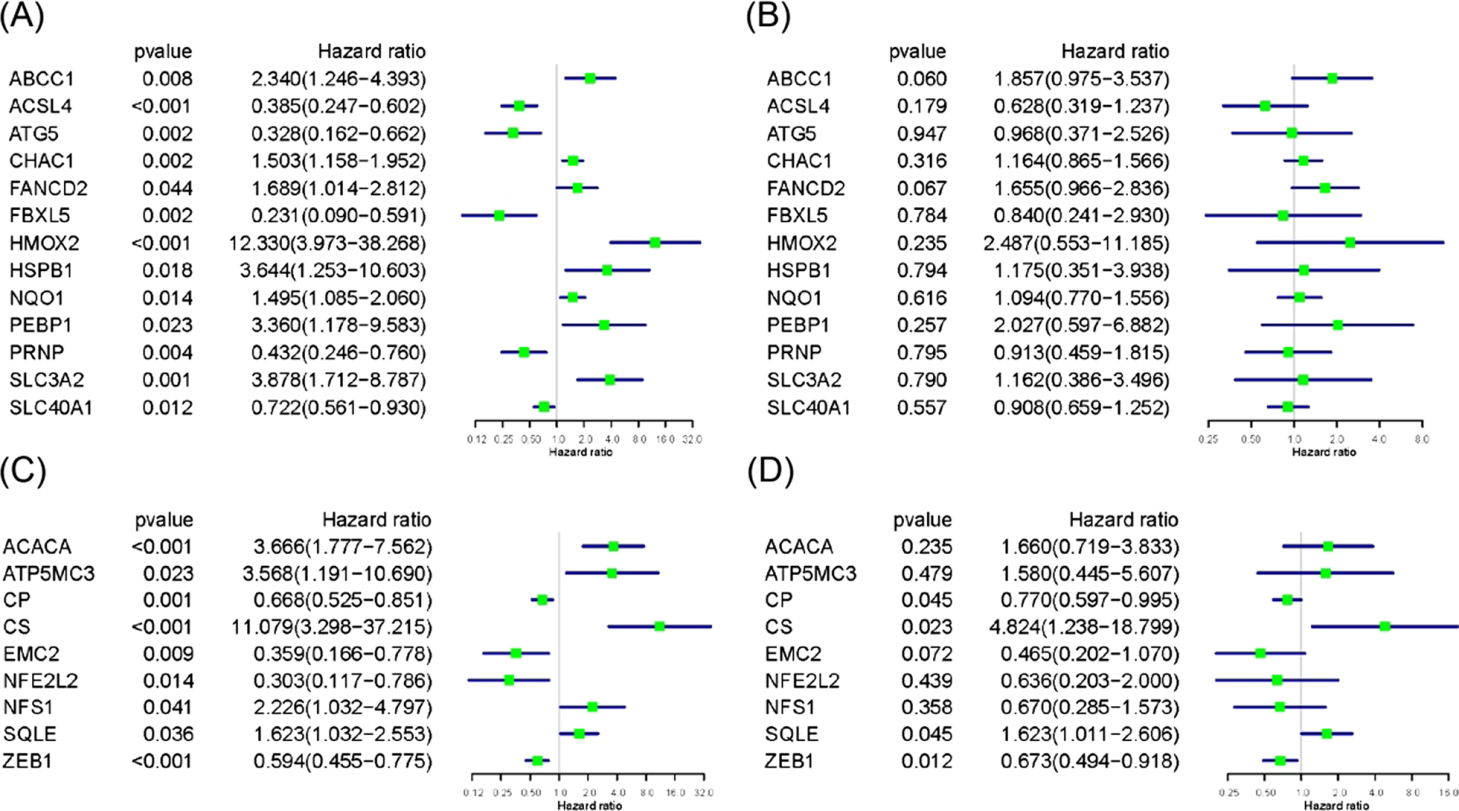
Independent prognostic analysis of each ferroptosis-related gene. **A**, **B** Univariate and multivariate Cox regression analysis of over-expressed ferroptosis-related genes. **C**, **D** Univariate and multivariate Cox regression analysis of low-expressed ferroptosis-related genes. CP and ZEB1 displayed independent prognostic ability in both analyses

In the group of highly expressed genes, although all genes had the independent prognostic ability in univariate Cox regression analysis, none of them can be realized with independent prognostic ability in multivariate Cox regression (Fig. 5A, B). In addition, there were 4 low-expressed ferroptosis-related genes (CP, CS, SQLE, ZEB1) in univariate and multivariate Cox regression analysis that can be regarded as independent prognostic genes (Fig. 5C, D). Because these genes were lowed-expressed in melanoma tissues, but Hazard ratio of CS and SQLE were on the right side of 1 in the forest map, we selected CP and ZEB1 for the next analysis.

### Evaluating prognostic value of CP and ZEB1 expression in melanoma patients

**Fig. 6:**
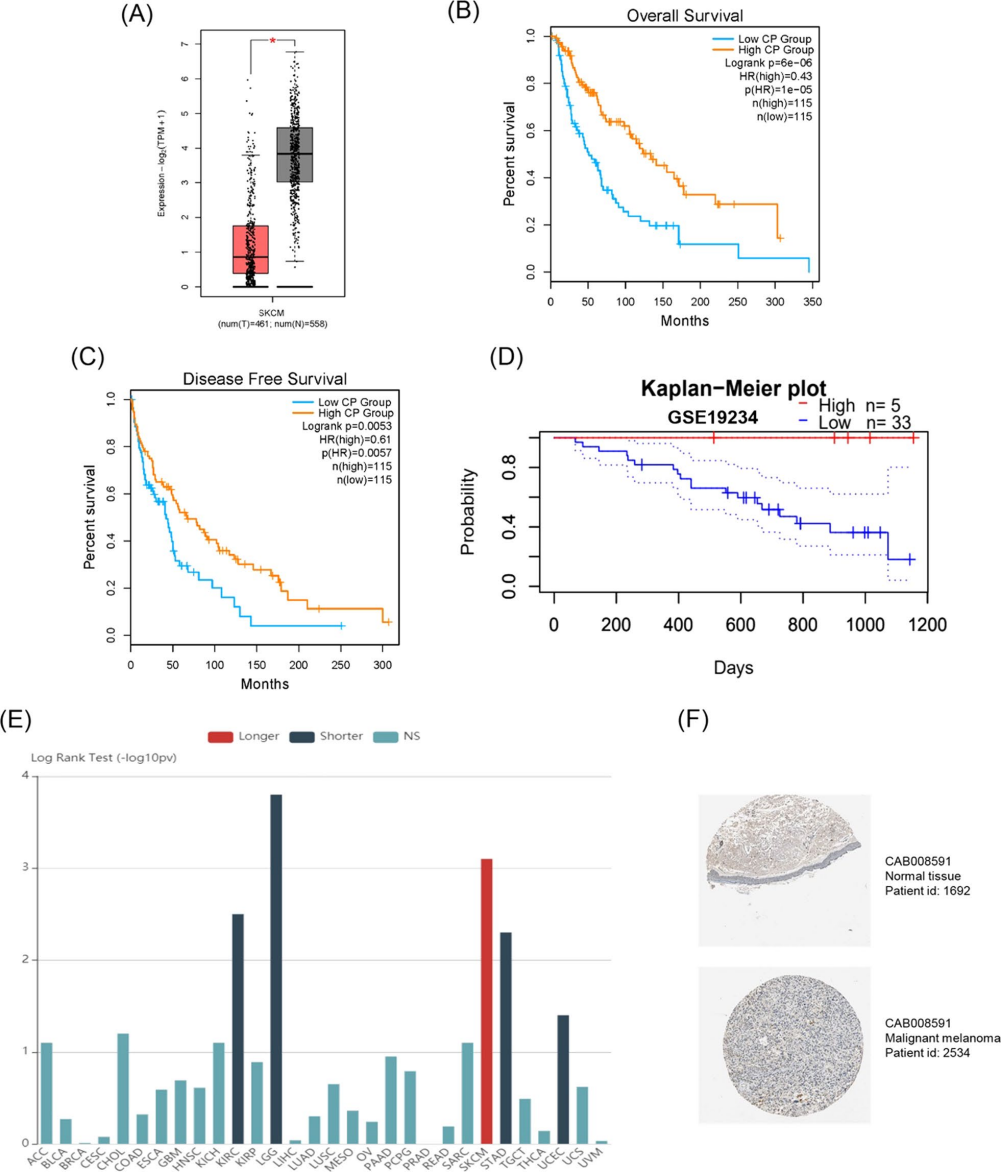
Verification of the low expression and effect of CP in melanoma in GEPIA, PrognoScan, TISIDB and HPA databases. **A** The expression of CP in tumor tissues and normal tissues in GEPIA database. **B**, **C** K-M curve of CP with OS and DFS in GEPIA database. **D** K-M curve of CP with OS in PrognoScan database. **E** Correlation between CP expression and survival time in all tumors in TISIDB database. **F** Immunohistochemical section of CP in HPA database

**Fig. 7:**
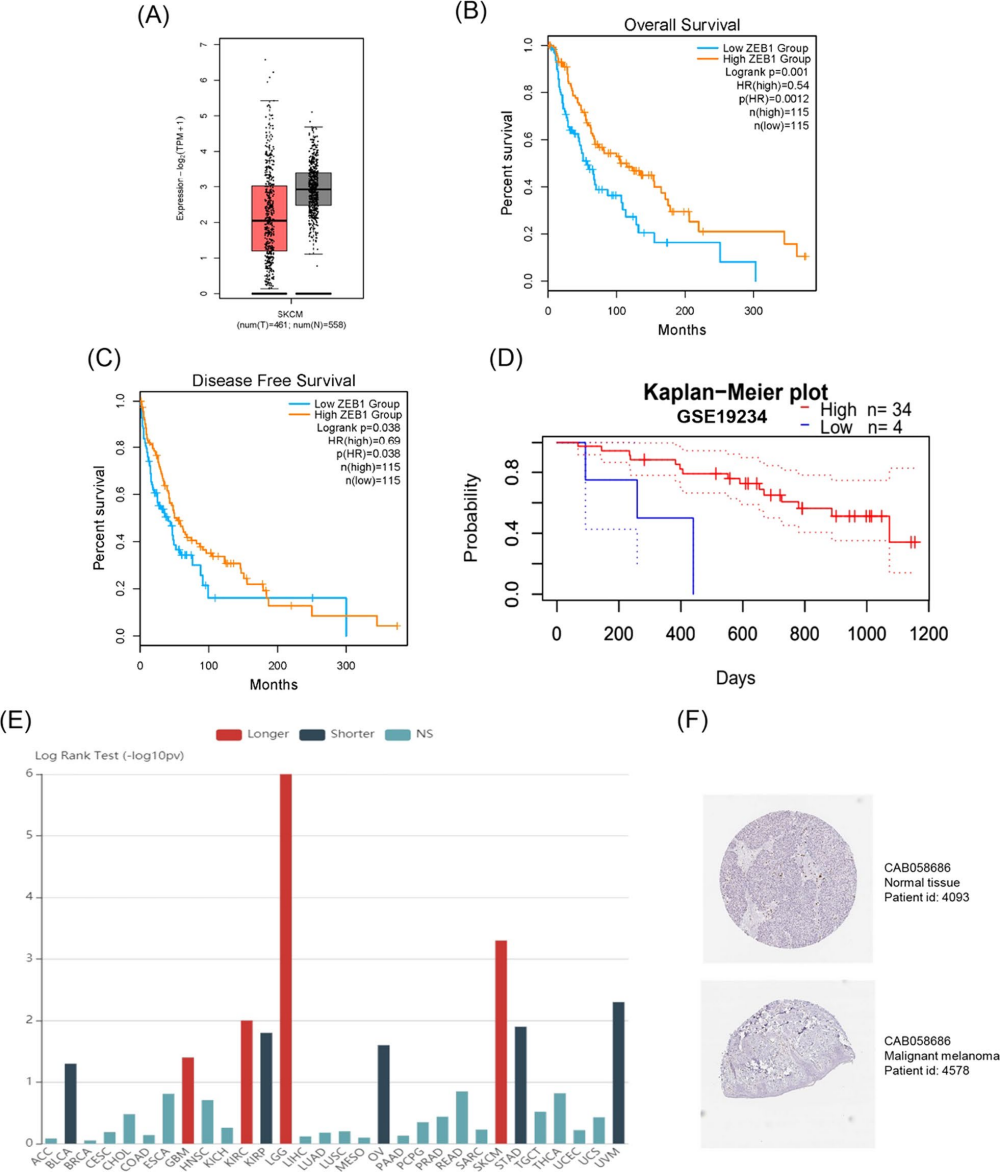
Verification of the low expression and effect of ZEB1 in melanoma in GEPIA, PrognoScan, TISIDB and HPA databases. **A** The expression of ZEB1 in tumor tissues and normal tissues in GEPIA database. **B**, **C** K-M curve of ZEB1 with OS and DFS in GEPIA database. **D** K–M curve of ZEB1 with OS in PrognoScan database. **E** Correlation between ZEB1 expression and survival time in all tumors in TISIDB database. **F** Immunohistochemical section of ZEB1 in HPA database

In GEPIA database, we again proved that CP was low expressed in 461 tumor samples and 558 normal samples (*P* < 0.05, Fig. 6A). Then we drew the K–M curves of CP in relation with OS and DFS in melanoma patients. Low expression of ZEB1 in melanoma patients was closely related to short OS and DFS (OS: *P* = 6e−06; DFS: *P* = 0.0053; Fig. 6B, C). In PrognoScan database, we utilized GEO matrix (GSE19234) to verify that we obtained the same results (Fig. 6D). In TISIDB database, surprisingly, we found that among all known tumors, the high expression of CP only showed a longer survival time in melanoma patients (Fig. 6E). Immunohistochemistry elucidated that CP was under-expressed in tumor tissues (Fig. 6F). Next, we performed the same analyses on ZEB1. The expression of ZEB1 in tumor tissues was lower than that in adjacent normal tissues (Fig. 7A), and the high expression of ZEB1 will promote longer OS and DFS (OS: *P* = 0.001; DFS: *P* = 0.038; Fig. 7B, C). Patients with low ZEB1 expression had a shorter survival time in the PrognoScan database (Fig. 7D). Similarly, ZEB1 patients had a longer survival time in the TISIDB database (Fig. 7E). Immunohistochemistry showed that ZEB1 was expressed slightly higher in normal tissues than in tumors (Fig. 7F).

### Genomic alternations and mutation survival analysis in melanoma patients

**Fig. 8:**
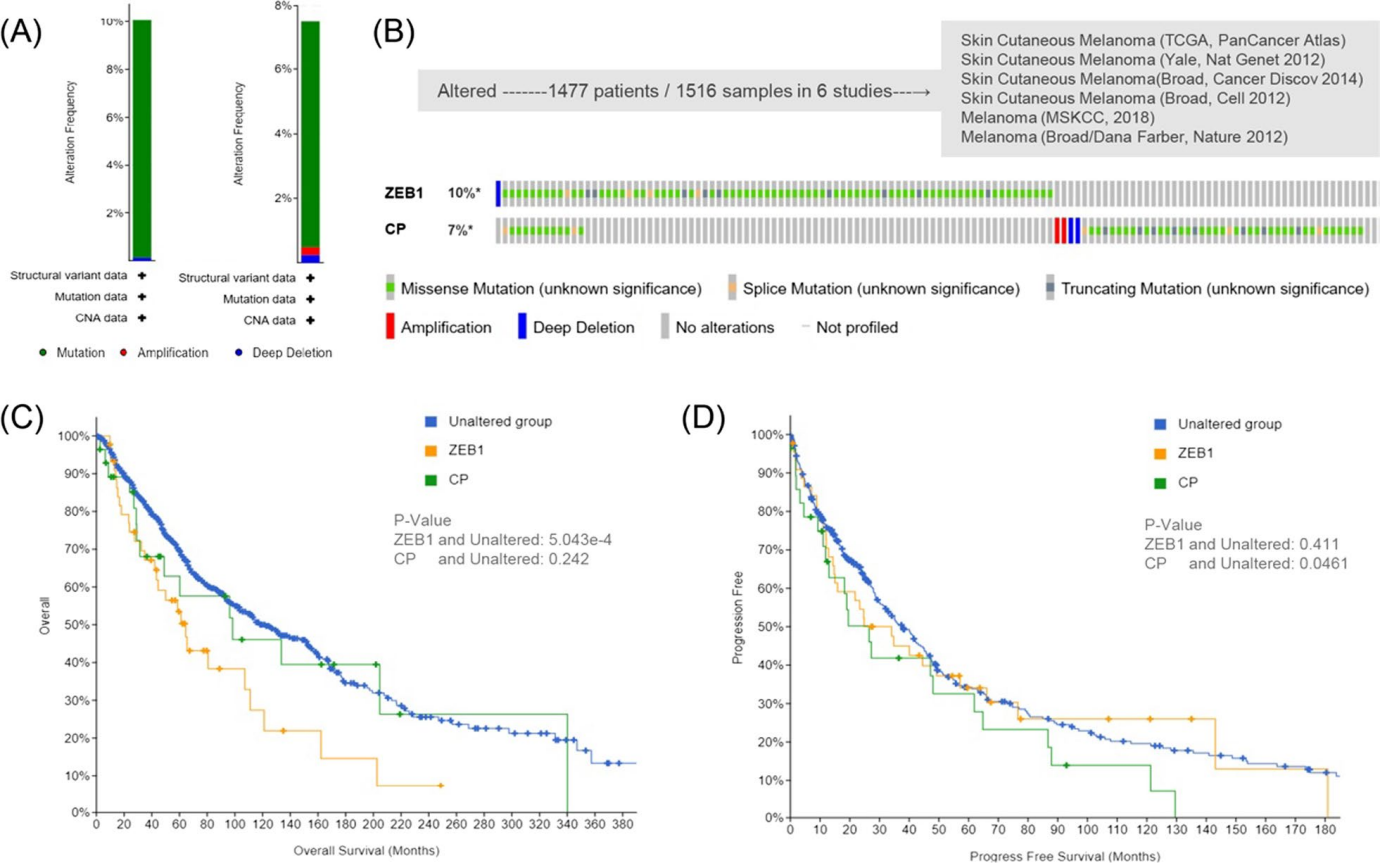
Genomic alternation of ZEB1 and CP and the effects of mutations on OS and progression-free survival (PFS) in the cBioPortal database. **A** Genomics alternation forms of ZEB1 and CP in melanoma, respectively. **B** Mutation frequency of ZEB1 and CP from 1516 melanoma samples in 6 studies. **C**, **D** K-M curves of OS and PFS in CP and ZEB1 mutation group and non-mutation group, respectively

Using the cBioPortal database, we analyzed the genetic alternations of ZEB1 and CP in 1516 samples from 1477 melanoma and skin melanoma patients. Two or more alternations were detected in the samples. The common manifestation of ZEB1 was depletion alterations, while amplification and depletion alterations were more common in CP (Fig. 8A). ZEB1 and CP were altered in 10% and 7% of the queried melanoma samples, respectively (Fig. 8B). Furthermore, we analyzed the relationship between gene mutation and survival status. It showed that the mutation of ZEB1 would lead to shorter OS in patients with melanoma (*P* < 0.001, Fig. 8C), while the progression survival time (PFS) of patients with the mutation of CP was significantly decreased (*P* < 0.05, Fig. 8D). This indicated that the mutation of ZEB1 may influence OS of melanoma patients and mutation of CP may be related to tumor progression.

### Tumor immune estimation resource (TIMER)

**Fig. 9:**
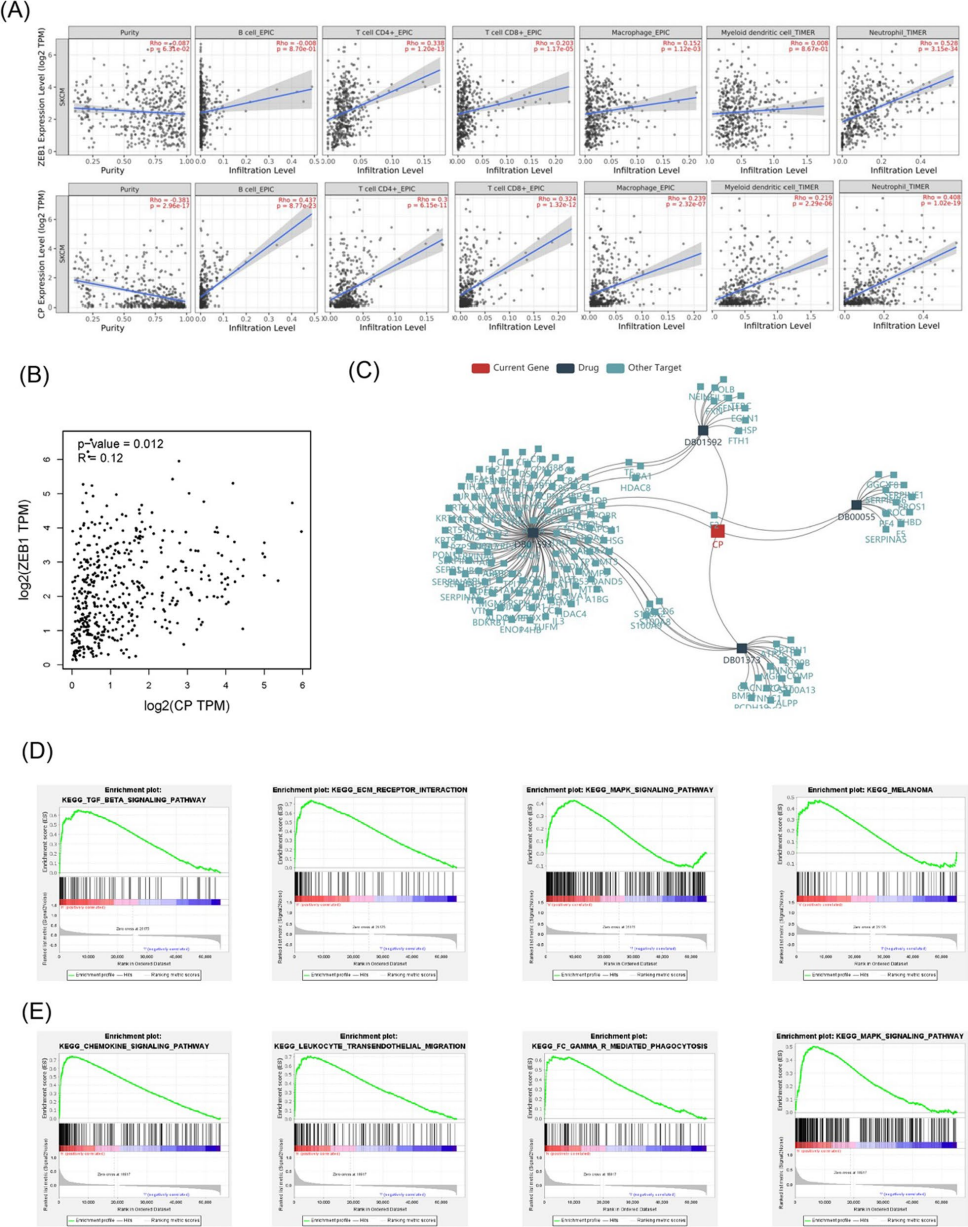
The relationship between two genes and immune cell infiltration and GSEA function enrichment analysis. **A** Correlation between genes and the infiltration of 6 immune cells is in the TIMER database. **B** The positive correlation of CP and ZEB1. **C** Network diagram of CP as a treatment target for 4 kinds of cancer drugs. **D**, **F** GSEA analysis of CP and ZEB1 illustrated that ZEB1 was related to cell proliferation, differentiation, and migration, while CP was associated with cytokine pathway and immune cell infiltration

Many studies had shown that immune infiltration in various tumor types is related to the patient's prognosis and treatment response [32]. We used the TIMER database to assess whether the expression of ZEB1 and CP were correlated with the level of immune infiltration. In the melanoma samples, ZEB1 level was positively related to CD4+ T cells (R = 0.338, *p* = 1.20e−13), CD8+ T cells (R = 0.203, *p* = 1.17e−5), macrophages (R = 0.152, *p* = 1.12e−03), dendritic cells (R = 0.008, *p* = 8.67e−1) and neutrophils (R = 0.528, *p* = 3.15e−34). Similarly, the level of CP had a positive correlation with B cells (R = 0.437, *p* = 8.77e−23), CD4+ T cells (R = 0.3, *p* = 6.15e−11), CD8+ T cells (R = 0.324, *p* = 1.32e−12), macrophages (R = 0.239, *p* = 2.32e−07), dendritic cells (R = 0.219, *p* = 2.29e−06) and neutrophils (R = 0.408, *p* = 1.02e−19) (Fig. 9A). Then, in order to investigate whether ZEB1 and CP were related, we analyzed the correlation between the two genes in the GEPIA database. It showed that they were positively correlated (R = 0.12, *p* = 0.012) (Fig. 9B). Next, we explored the therapeutic-related role of CP and ZEB1 in the TISIDB database. CP was currently known as the target of several drugs, while ZEB1 as tumors therapeutic drug target had not yet been recorded, which may require further research (Fig. 9C).

### Gene set enrichment analysis (GSEA)

To clarify the molecular mechanism of ZEB1 and CP, we divided the 471 tumor patients in TCGA into high- and low-risk groups. We employed GSEA to compare the high-risk and low-risk groups. The KEGG pathways enriched in the high-risk group of ZEB1 including TGF-b, ECM receptor, MAPK, melanoma, and other signal pathways. This indicated that ZEB1 may be involved in cellular functions such as proliferation, apoptosis, differentiation, adhesion, and migration (Fig. 9D). KEGG pathways including chemokine, leukocyte transendothelial migration, Fc gamma R-mediated phagocytosis, MAPK, and other signal pathways enriched in the high-risk group of CP. This suggested that CP was closely related to tumor immunity, cellular proliferation, and migration (Fig. 9E).

## Discussion

In this work, our goal is to analyze the role of iron metabolism and ferroptosis-related genes in the prognosis of melanoma patients comprehensively through high-throughput array technology. Although there are some reported ferroptosis-related signature and some biomarkers in melanoma and other tumors, including uveal melanoma [20, 21]. However, given the heterogeneity among different tumors, for example, although CM and uveal melanoma originate from the same melanocyte lineage, they differ in their cellular alterations, such as, somatic mutation profiles, tumor mutational burden, etc., and differ in pathogenesis and biological behavior, with different transfer pathways and tropisms [33, 34]. Therefore, it may be more specific to construct a prognostic model for cutaneous melanoma. When processing data, in view of the small size of non-tumor samples in the TCGA cohort, we combined with normal samples in the GTEx database to systematically analyze the differential expression of iron death-related genes in melanoma. Differential genes with prognostic ability are divided into high expression and low expression groups. After LASSO regression analysis, the high expressed-genes were used to construct a 10-gene (ATG5, CHAC1, FANCD2, FBXL5, HMOX2, HSPB1, NQO1, PEBP1, PRNP, SLC3A2) prognostic signature in the TCGA cohort, which was validated by the GEO database, proving an excellent ability to predict prognosis. The AUC values of the ROC curves in our model ranged from 59 to 79% (Total AUC: 59–63%; I/II stage AUC: 59–67%; III/IV stage AUC: 63–79%), and the ROC curves of the prognostic models constructed by Gao et al. and Gao et al. showed similar results in AUC values (AUC: 63–72% and 52–70%, respectively) [35, 36]. Although its value was not as good as that of Jonsson G et al. and Nsengimana et al., the ferroptosis-related prognostic signature we constructed was still novel and outperformed the other similar ferroptosis-related prognostic models reported [37–39]. The following univariable and multivariable Cox regressions displayed that prognostic signature can be regarded as an independent prognostic factor. In addition, the nomogram we constructed also revealed a good prognostic ability. GO and KEGG enrichment analyses elucidated that these genes were related to epidermal growth, cell proliferation, differentiation, and apoptosis. In the ssGSEA analysis, there were many differences in immune cells and immune functions, indicating that these genes were related to immunity. Genes in ferroptosis-related prognostic signature can be divided into three categories, including genes that promote ferroptosis sensitivity (ATG5, CHAC1, PEBP1), suppress ferroptosis sensitization genes (FANCD2, HSPB1, NQO1, SLC3A2), and iron metabolism related genes (FBXL5, HMOX2, PENP). However, these genes were all upregulated in cutaneous melanoma tissues and were associated with poor prognosis in the current study, and their mechanical in the prognosis of cutaneous melanoma patients remains to be elucidated. ATG5 (autophagy-related 5) can facilitate ferroptosis by regulating ferritin degradation, and knockdown of ATG5 can attenuate the effect of ferroptosis inducers, decrease intracellular ferrous iron levels and lipid peroxidation [40]. CHAC1 (Glutathione-Specific Gamma-Glutamylcyclotransferase 1) is involved in the RIP1/RIP3-MLKL pathway and contributes to cystine starvation-induced cell death. Knockdown of CHAC1 rescues cystine starvation-induced decrease in glutathione (GSH) levels and cell death [41]. And the study found that the level of CHAC1 protein was significantly up-regulated in cells treated with ferroptosis inducers [42]. FANCD2 (Fanconi anemia complementation group D2) is a nuclear protein involved in DNA repair, and its expression level is up-regulated when ferroptosis inhibitor is added, suggesting that it has a negative role in ferroptosis and can be further utilized to reduce the side effects of ferroptosis treatment drugs [40]. FBXL5 (F-box and leucine-rich repeats protein 5) maintains intracellular iron homeostasis mainly by regulating the degradation of IRP2 (iron regulatory protein 2) [20]. HMOX2 is an isoenzyme of heme oxygenase, which catalyzes the degradation of heme into ferrous iron and other substances [43]. Activation of HMOX2 by oncogenic BRAF promotes the increase of melanospheres in melanoma [44]. HSPB1 (heat shock protein beta-1) can be mediated by Protein kinase C, which in turn plays a negative regulatory role in ferroptosis [45]. NQO1 (NAD(P)H quinone dehydrogenase 1) is not beneficial to ferroptosis and is closely related to the tumor immune microenvironment [9, 46]. PEBP1 (phosphatidylethanolamine-binding protein 1) is a scaffold protein inhibitor of the protein kinase cascades, and can promote ferroptosis by combining with 15-lipoxygenases (15-LO) to generate hydroperoxy-PE [47]. PRNP (The prion protein) is involved in many cell biological processes and is associated with the prognosis of many tumors [6, 40]. It is mainly involved in iron metabolism and affects cellular oxidative stress by affecting the ERK pathway [48]. SLC3A2 has been shown to be associated with radiotherapy resistance in many tumors, and overexpression of SLC3A2 leads to worse prognosis by promoting tumor development and reducing apoptosis [49, 50].

On the other hand, we conducted analyses on low-expressed genes with independent prognostic value. We used a variety of databases to verify their low expression in melanoma and draw a survival curve, which once again proved their independent prognostic ability and potential effect. In addition, we explored the genetic mutations of these two genes and drew survival curves of the mutant group and the non-mutant group. It showed that the mutation of ZEB1 could cause a significant decrease in the OS in melanoma patients, and mutation of CP was closely related to the progression of the tumor. Next, we discussed the immune infiltration level of ZEB1 and CP in melanoma. These two genes were positively correlated in the 6 immune cells, and there was also a positive correlation between ZEB1 and CP. We further found that CP had been used as a target for four tumor drugs, while ZEB1 did not have targeted drugs yet. Therefore, our study also provided a new perspective for future drug research and development. Finally, GESA was used to analyze these two genes. The results showed that ZEB1 was closely related to cell proliferation, differentiation, apoptosis, adhesion, and metastasis, and CP was closely related to cytokine release and immune-related pathways. CP (Ceruloplasmin) is involved in the peroxidation of Fe (II) transferrin to Fe (III) transferrin. Although CP has increased expression in lung cancer [51], cholangiocarcinoma [52] and other tumors in previous studies, Zhu et al. found that the decline in CP expression indicated a poor prognosis for the patients in adrenocortical carcinoma [53]. In our study, the expression level of CP in melanoma is presumably decreased in tumor tissues and the PFS of melanoma patients with CP mutation is significantly reduced. However, the study of CP in melanoma tumors has not been fully conducted, which provides ideas for the next research. ZEB1 (Zinc Finger E-Box Binding Homeobox 1) is an important gene that can promote epithelial-mesenchymal transition (EMT). The low expression of ZEB1 can impede EMT, reducing the sensitivity of cells to ferroptosis, which will weaken the ability to resist cancer [54].

There are still some limitations in our study. First, we utilized retrospective data from public databases to construct and verify our prognostic model; Second, we applied only a single biomarker to construct a prognostic model, which may exclude other important genes of melanoma. Third, compared with some previous studies on prognostic markers, our study lacks a more rigorous design, such as more specific stages, intervention factors, and so on [38, 39, 55]. Overall, our study focuses on ferroptosis and iron metabolism and defines a 10-prognostic gene model related to iron death and this model has a good predictive ability. More importantly, we find that CP and ZEB1 have special effects in melanoma, which also provides a new insight for future research exploring molecular mechanism and drug treatment in melanoma. Research on the correlation of melanoma with ferroptosis and immunity is worthy of further study to explore potential molecular mechanisms and improve prognosis.

## Conclusions

In conclusion, we identified 22 ferroptosis-related DEGs associated with the prognosis of melanoma patients, constructed a 10-gene prognostic signature, and drew a nomogram to predict the prognosis of melanoma patients. Then we discovered and analyzed the effects of CP and ZEB1 in melanoma patients. The clusters showed significant associations with cell proliferation, differentiation, adhesion, migration, and immune function. These biomarkers can properly predict the survival time of melanoma patients. Further studies should be performed to explore the precise role of these genes in cutaneous melanoma.

## Supplementary Information

**Additional file 1: Supplementary Figure 1.** Prognostic analysis of a 10-biomarker signature in melanoma patients at different stages in TCGA matrix. **Supplementary Figure 2.** Prognostic analysis of a 10-biomarker signature metastatic group and primary group in TCGA matrix. **Supplementary Figure 3.** ROC curves for disease prognosis prediction with different clinical covariates and risk scores.

**Additional file 2: Table S1.** Ferroptosis and iron metabolism-related genes.

**Additional file 3: Table S2.** The annotated gene set file used in ssGSEA.

**Additional file 4: Table S3.** 76 differentially expressed ferroptosis genes and their calculated values.

**Additional file 5: Table S4.** Univariate regression values for 22 intersection genes.

**Additional file 6: Table S5.** Differentially expressed genes in high and low risk groups.

**Additional file 7: Table S6.** Results of GO enrichment analysis of differential genes in high and low risk groups.

**Additional file 8: Table S7.** Results of KEGG enrichment analysis of differential genes in high and low risk groups.

## Data Availability

The raw datasets used during the present study can be downloaded from The Cancer Genome Atlas (https://portal.gdc.cancer.gov/projects/), UCSC Xena (https://xenabrowser.net/datapages/) and GSE65904 (https://www.ncbi.nlm.nih.gov/geo/query/acc.cgi?acc=GSE65904). All RNA-seq data and GSE65904 expression profile microarray datasets are open access and can be downloaded directly from the corresponding database without any login account. Ferroptosis and iron metabolism-related genes were obtained from KEGG (https://www.genome.jp/kegg/pathway.html), Reactome (https://reactome.org/), and AmiGo2(http://amigo.geneontology.org/amigo). Some results were analyzed online and aggregated directly from multiple databases without relevant accession numbers. Direct web links of datasets: STRING, https://string-db.org/, version 11.0; GeneMANIA, http://www.genemania.org; Metascape, http://metascape.org; Gene Expression Profiling Interactive Analysis (GEPIA), http://gepia.cancer-pku.cn/index.html; PrognoScan, http://dna00.bio.kyutech.ac.jp/PrognoScan/index.html, cBioPortal, http://www.cbioportal.org; TISIDB, http://cis.hku.hk/TISIDB/; TIMER, https://cistrome.shinyapps.io/timer/; The Human Protein Atlas (HPA), https://www.proteinatlas.org/.

## Footnotes

1 https://portal.gdc.cancer.gov/projects.

2 https://xenabrowser.net/datapages/

3 http://www.ncbi.nlm.nih.gov/geo/.

4 https://www.genome.jp/kegg/pathway.html.

5 https://reactome.org/

6 http://amigo.geneontology.org/amigo.

7 https://string-db.org/, version 11.0.

8 http://www.genemania.org.

9 https://metascape.org/.

10 http://gepia.cancer-pku.cn/index.html.

11 http://dna00.bio.kyutech.ac.jp/PrognoScan/index.html.

12 http://www.cbioportal.org.

13 http://cis.hku.hk/TISIDB/.

14 https://cistrome.shinyapps.io/timer/.

15 https://www.proteinatlas.org.
