# Supplementary material for: Comprehensive analysis of ferroptosis-related genes and prognosis of cutaneous melanoma
Source: BMC Med Genomics. 2022 Mar 1;15:39. doi: 10.1186/s12920-022-01194-z (PMC8886785; doi:10.1186/s12920-022-01194-z)
Supplement: Supplementary file 1 — Additional file 1: Supplementary Figure 1. Prognostic analysis of a 10-biomarker signature in melanoma patients at different stages in TCGA matrix. Supplementary Figure 2. Prognostic analysis of a 10-biomarker signature metastatic group and primary group in TCGA matrix. Supplementary Figure 3. ROC curves for disease prognosis prediction with different clinical covariates and risk scores. [file 12920_2022_1194_MOESM1_ESM.docx]

**Supplementary materials Figures**

**Supplementary materials Figure 1 |** Prognostic analysis of a 10-biomarker signature in melanoma patients at different stages in TCGA matrix.

**Supplementary materials Figure 2 |** Prognostic analysis of a 10-biomarker signature metastatic group and primary group in TCGA matrix.

**Supplementary material Figure 3 |** ROC curves for disease prognosis prediction with different clinical covariates and risk scores


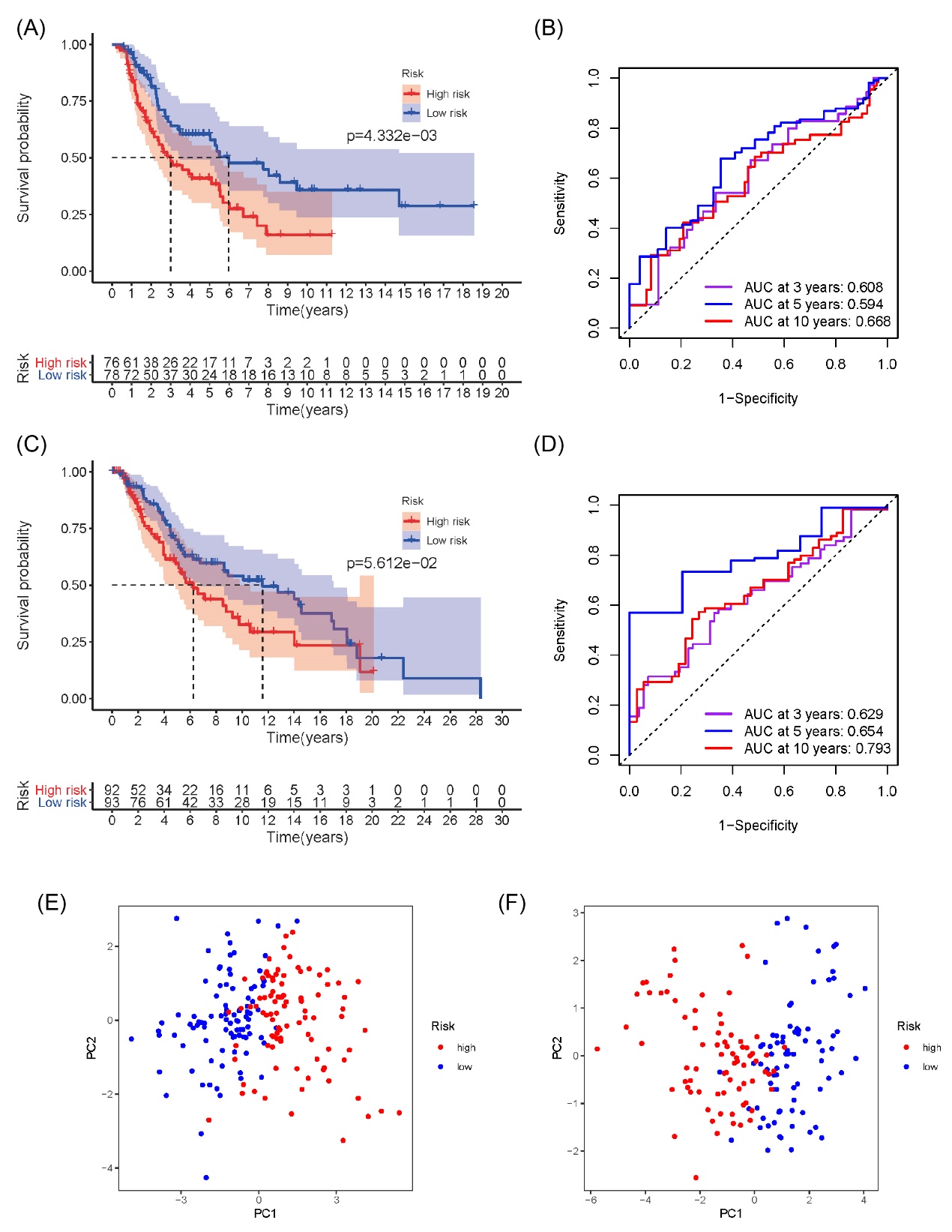
 **Supplementary materials Figure 1 |** Prognostic analysis of a 10-biomarker signature in melanoma patients at different stages in TCGA matrix. **(A)** and **(C)** Kaplan-Meier curves for two different-risk melanoma patient groups in stage I/II group and stage III/IV group, respectively. **(B)** and **(D)**the ROC curves of 3, 5, and 10 years of survival rates using 10 genes prognostic model in stage I/II group and stage III/IV group, respectively. **(E)** and **(F)** PCA plots of the melanoma patients at stage I/II and stage III/IV, respectively.


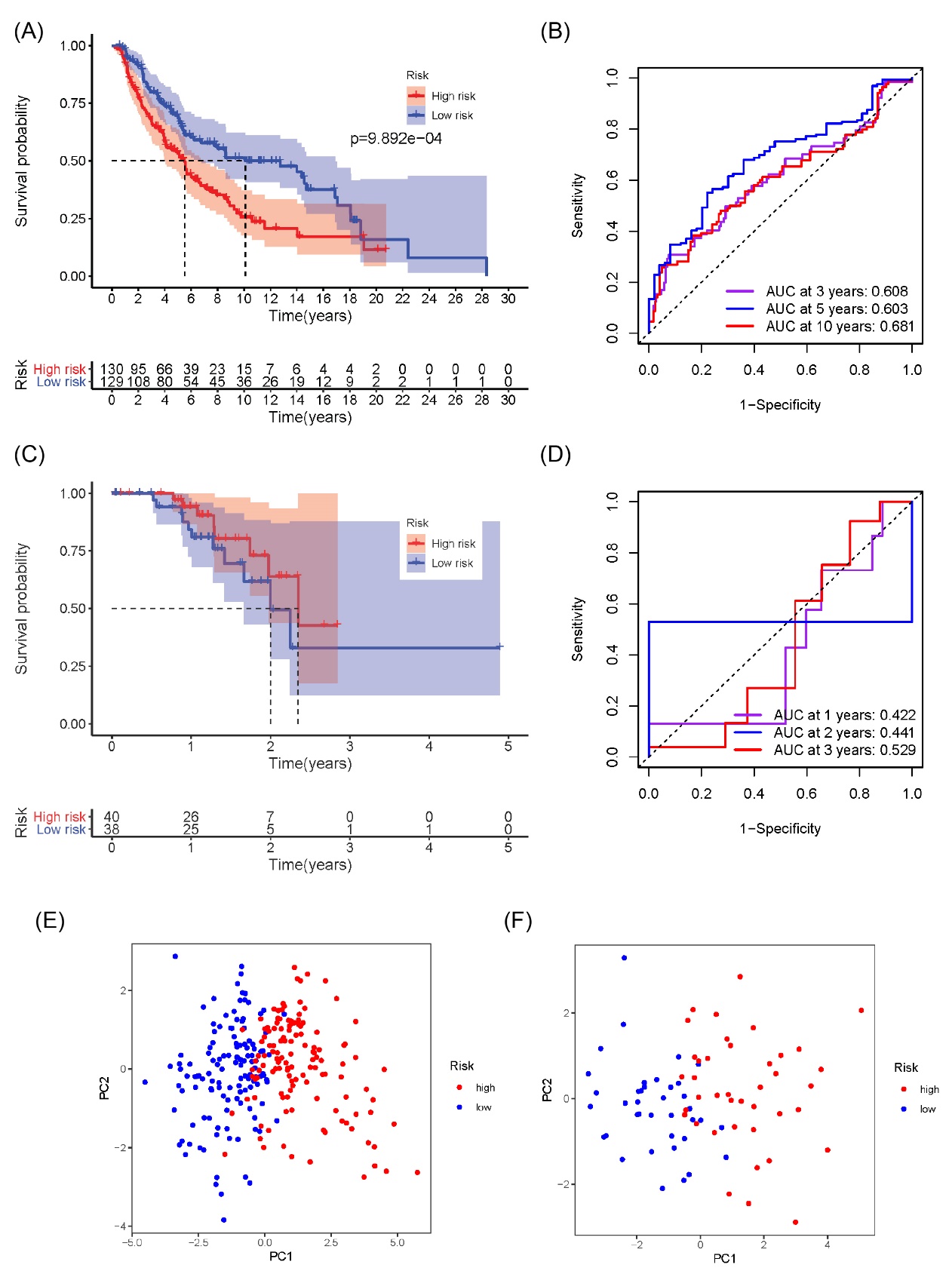
 **Supplementary materials Figure 2 |** Prognostic analysis of a 10-biomarker signature metastatic group and primary group in TCGA matrix. **(A)** and **(C)** Kaplan-Meier curves for two different-risk melanoma patient groups in metastatic group and primary group, respectively. **(B)** the ROC curves of 3, 5, and 10 years of survival rates using 10 genes prognostic model in metastatic group. **(D)** the ROC curves of 3, 5, and 10 years of survival rates using 10 genes prognostic model in primary group. **(C)** and **(D)** PCA plots of the melanoma patients in metastatic group and primary group, respectively.


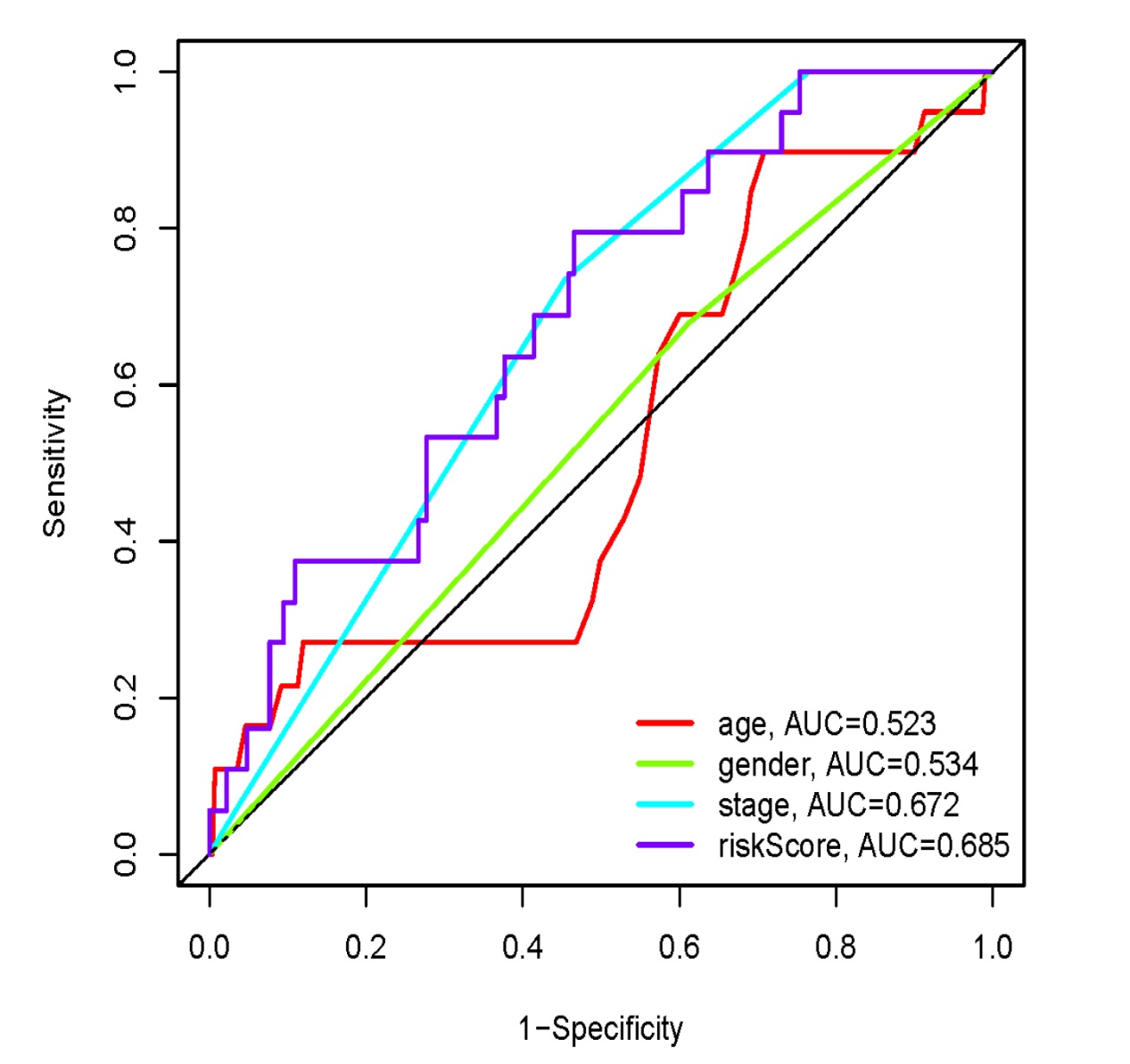


**Supplementary material Figure 3 |** ROC curves for disease prognosis prediction with different clinical covariates and risk scores

**Supplementary materials Tables Content**

**Table S1:** Ferroptosis and iron metabolism-related genes.

**Table S2.** The annotated gene set file used in ssGSEA.

**Table S3:** 76 differentially expressed ferroptosis genes and their calculated values.

**Table S4:** Univariate regression values for 22 intersection genes.

**Table S5:** Differentially expressed genes in high and low risk groups.

**Table S6:** Results of GO enrichment analysis of differential genes in high and low risk groups.

**Table S7:** Results of KEGG enrichment analysis of differential genes in high and low risk groups.
